# Pitfalls in the embolisation of a thyrocervical trunk bleeding: a case report

**DOI:** 10.1186/s13037-020-00244-8

**Published:** 2020-05-06

**Authors:** Luca Perrucci, Monica Graziano, Zairo Ferrante, Elisabetta Salviato, Aldo Carnevale, Roberto Galeotti

**Affiliations:** 1 Department of Interventional and Diagnostic Radiology, Arcispedale Sant’Anna, Ferrara, Italy; 2 Section of Diagnostic Imaging, Department of Morphology, Surgery and Experimental Medicine, University of Ferrara, Ferrara, Italy; 3 Azienda Ospedaliero-Universitaria - Nuovo Sant’Anna Hospital, via A. Moro 8, Cona, Ferrara, Italy; 4 Radiology Department, University Radiology Unit, Sant’Anna University Hospital, Ferrara, Italy

**Keywords:** Emergency radiology, Embolisation, Head/neck, Haemorrhage, Trauma

## Abstract

**Introduction:**

An intrathoracic bleeding from the thyrocervical branch is not common in blunt trauma, but an interventional radiologist should be aware of the risks in order to prevent complications.

**Case presentation:**

A 30-year-old male presented with a right pneumo-haemothorax due to active bleeding revealed at contrast-enhanced CT, as a consequence of a fall occurred in the previous week. The patient was treated with endovascular embolisation in an angiographic room with coils placement, since the right thyrocervical artery was found to be supplying the pneumo-haemothorax. A radiculo-medullary branch rose from the thyrocervical trunk, impeding the proximal embolization with microparticles and needing selective isolation of the bleeding artery with the catheter to avoid spinal cord injuries. The treatment had a successful result and the following CT control showed signs of recovering, without any complication.

**Conclusion:**

Our paper presents a rare contingency, warning the operator to bear in mind the presence of arteries feeding the spinal cord. This crucial detail precludes the use of microparticles embolisation to prevent neurologic sequelae, whereas the use of endovascular coils for embolization should be mandatory. Moreover, this case reminds that the post-traumatic bleeding deriving from a cervical trauma may also occur later.

## Background

The thyrocervical trunk is an ascending branch coming off the superior wall of the subclavian artery, laterally and in parallel to the vertebral artery. It divides into four branches: the inferior thyroid artery, the ascending cervical artery, the suprascapular artery and the transverse cervical artery. A noticeable anatomic variability of these vessels is known and therefore should be expected from the interventional radiologist. An important aspect to hold in consideration is that the anterior spinal artery, which runs longitudinally in the anterior median groove of the spinal cord, receives radiculo-medullary arteries arising from the neck arteries. Even the thyrocervical trunk emits feeding collaterals to the anterior spinal artery, usually from the ascending cervical branch; furthermore it may be linked to the brainstem through collaterals to the vertebral arteries. These anastomoses imply the risk of neurological sequelae after the embolisation of the thyrocervical trunk. Pérez-García et al.(2019) describes in a wide case series this potential complication in the presence of spinal collateral of thyrocervical artery in endovascular embolisation procedures performed as preoperative treatment for the resection of tumours or as therapy for recurrent haemoptysis, aneurysmal bone cyst and post-traumatic active-bleeding using both microspheres and coils [1]. A paper of Tanizaki et al. (2012) presents a similar case of a consistent haemothorax for thyrocervical trunk bleeding in coincidence with a fall treated with coils, but the warning of spinal branches was not mentioned [2] as in an other work where microparticles were used for its embolisation [3].

To the best of our knowledge, several cases of thyrocervical bleeding have been reported after invasive procedures, traumatic accidents, infective and vascular diseases with an unexpected association with the neurofibromatosis-1; all cases were treated successfully with endovascular coils, suggesting the value of this interventional approach [4–7]. As contrary, we found a case of a woman treated for haemoptysis with embolisation of the thyrocervical branch through microspheres that led to a spinal cord infarct [8]. In the literature, there has also been outlined further possible anastomoses such as between the thyrocervical trunk and the coronaries, both as congenital, and after by-pass surgery [9–11] or with the aortic arch [12]; therefore it is reasonable to suppose that in these cases an unselective embolization might potentially led to myocardial infarction or other fatal complications.

The embolisation can be wide or local, according to the vascular territory involved; moreover it can be permanent or temporary according to the embolic agent [13]. Temporary agents include Gelfoam, while permanent agents include metallic coils and embolization particles [14]. Scientific literature supports that the thyrocervical trunk can be embolised, but microparticles should be avoided unless the presence of communications with spinal or vertebral arteries is excluded. The latter are less challenging and more rapidly applied in the context of an emergency, but coils are preferred to preserve organ functions. The clinical scenario and the vascular variants may also affect the operative planning: a wide embolisation with particles is an easier and faster procedure when the operator knows it would be safe [13]. Moreover, there is no conclusive scientific evidence on the best approach for pneumo-haemothorax [15].

## Case presentation

Our patient came to the Emergency Department complaining of a rapid onset of back pain and dyspnea, following a fall on his back the week before during a circus performance. Upon arrival, the patient was tachycardic (118 bpm), euthermic and eupnoeic with normal blood pressure. Nothing in particular emerged from the familiar, habits and historical anamnesis. At the physical examination, the breath sound at the e lung base was absent. After fainting, the patient presented asthenia and stupor.

**Fig. 1:**
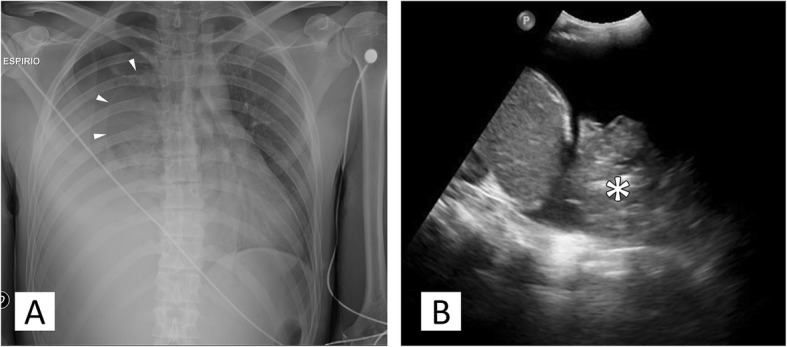
Chest X-Ray and Ultrasound. On the left picture (**a**), the supinous chest-X ray in expirium shows a right pneumothorax, suggested by the radiolucency of the apex and the collapsed the lung parenchyma (arrowheads pointing the visceral pleural line), while the radiopacity at right lung base shows the presence of pleural effusion. As a consequence, a slight controlateral mediastinal shift is present. The right lung effusion was confirmed with the ultrasound examination, revealing a dishomogenous hyperechoic material settled in the posterior pleural cavity compatible with a huge blood clot (asterisk) as illustrated in the figure on the right (**b**)

**Fig. 2:**
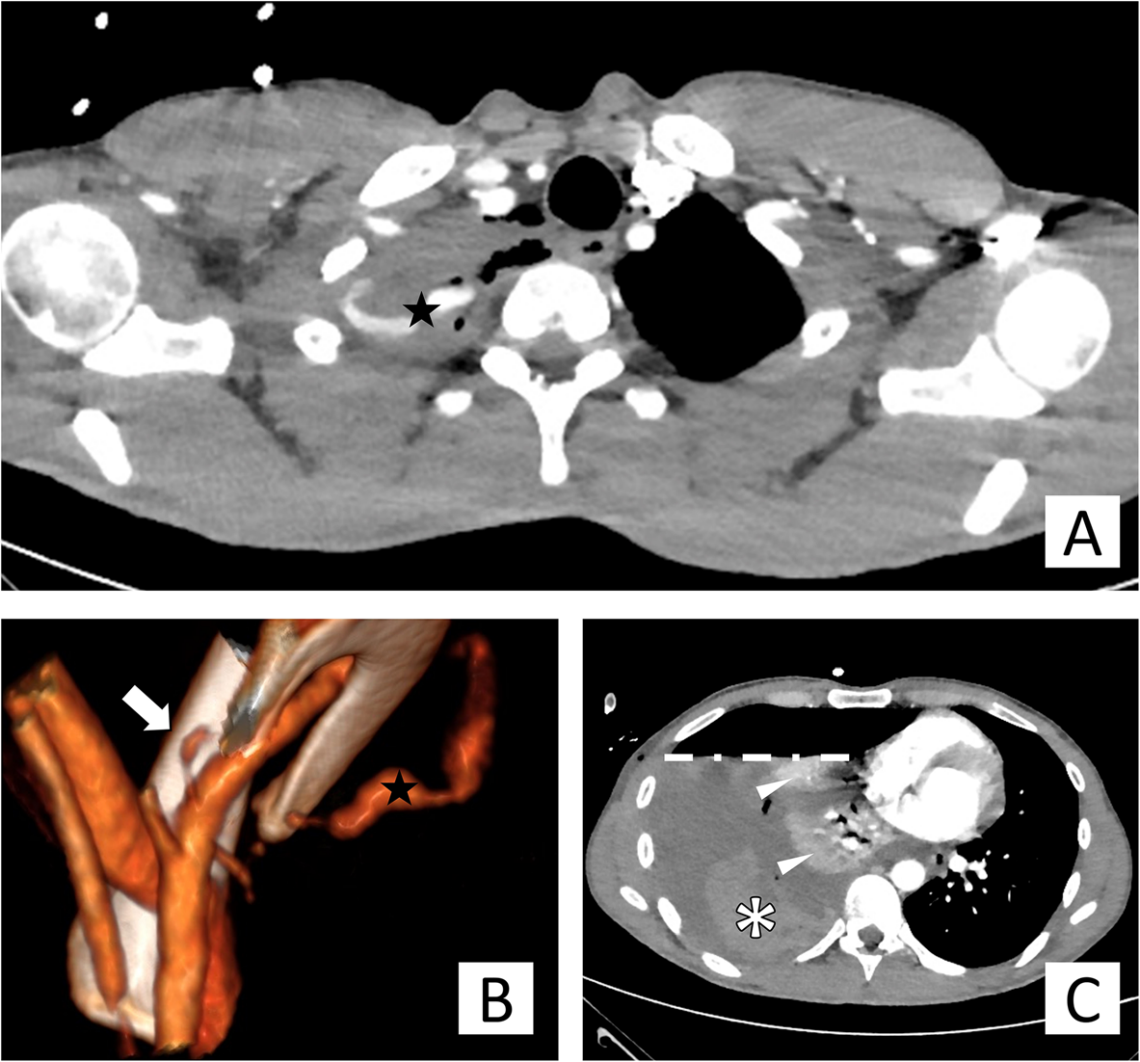
CT scan with contrast medium and Volume rendering of the active bleeding. The Axial CT scan (**a**) shows the blushing of contrast media in the right lung apex (star) that crosses into a blood clot. The volume rendering reconstruction (**b**) with a back view of the subclavian artery highlights its passage between the clavicle and the first rib. The three-dimensional reconstruction shows also, two vessels arising from the superior side of the artery, the thick white arrow points the thyrocervical trunk while the star indicates the outflow of contrast media. In the CT slice (**c**) are detectable: the right lung collapse (arrowheads), the presence of an air-fluid level (dashed line) with a dishomogeneous avascular irregular-shaped mass in the posterior pleural cavity as hallmark of a haemo-pneumothorax with huge coagulated blood (asterisk) and, lastly, the consequent mediastinal left-shifting

Chest X-ray, abdominal ultrasound and laboratory exams were performed. They showed signs of pneumo-haemothorax and lung collapse of the right hemithorax (Fig. 1). The critical clinical condition, the trauma history and the finding of anemia (Serum Haemoglobin level = 8,7 g/dL at admission) oriented to a post-traumatic pneumo-haemothorax with active bleeding. Right pneumo-haemothorax was treated with pleural drainage positioning and 2 l of haematic fluid were evacuated. A blood transfusion was performed in order to stabilise the patient before the Computed Tomography (CT) scanning with contrast media, which confirmed the diagnosis of bleeding revealing a haemorrhagic focus at the right lung apex with the suspicion of subclavian origin (Fig. 2).

**Fig. 3:**
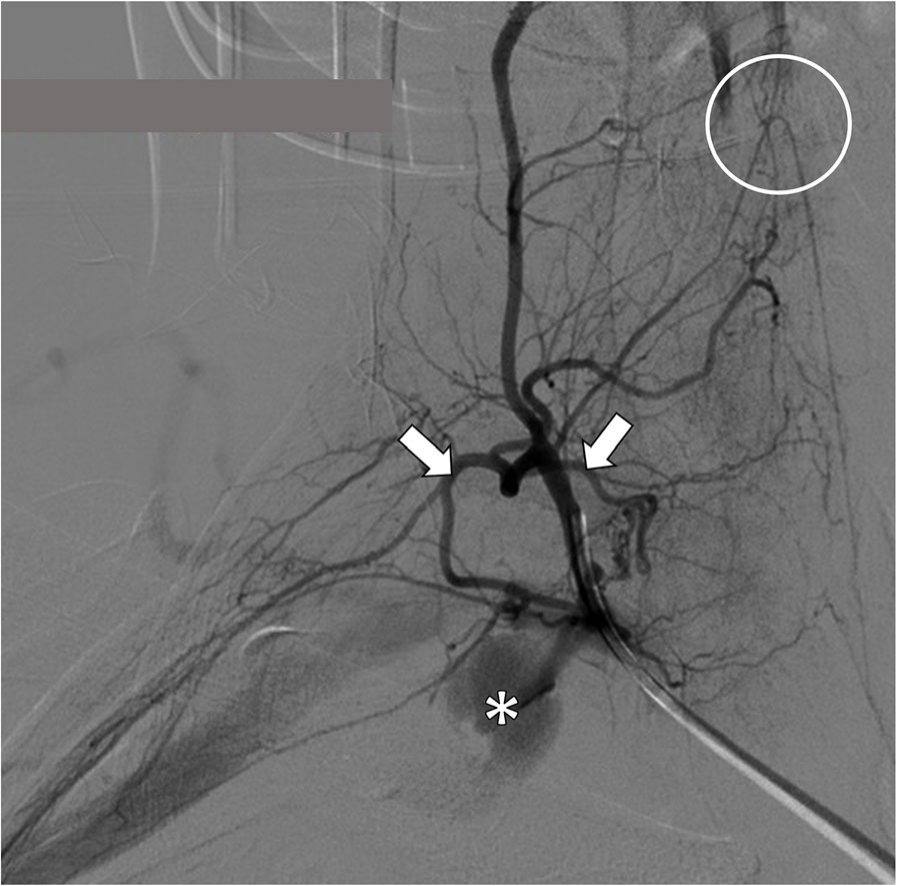
Digital Subtractive Angiography and operative planning. The picture taken with digital subtractive angiography shows the extravasation of blood from the thyrocervical trunk (asterisk) and the branch feeding the anterior mid-line artery of the spinal cord (white circle) that led to slide the catheter tip forward in order to reach a selective embolisation of the bleeding branches. The two vessels supplying the haematoma are highlighted with the white arrows

**Fig. 4:**
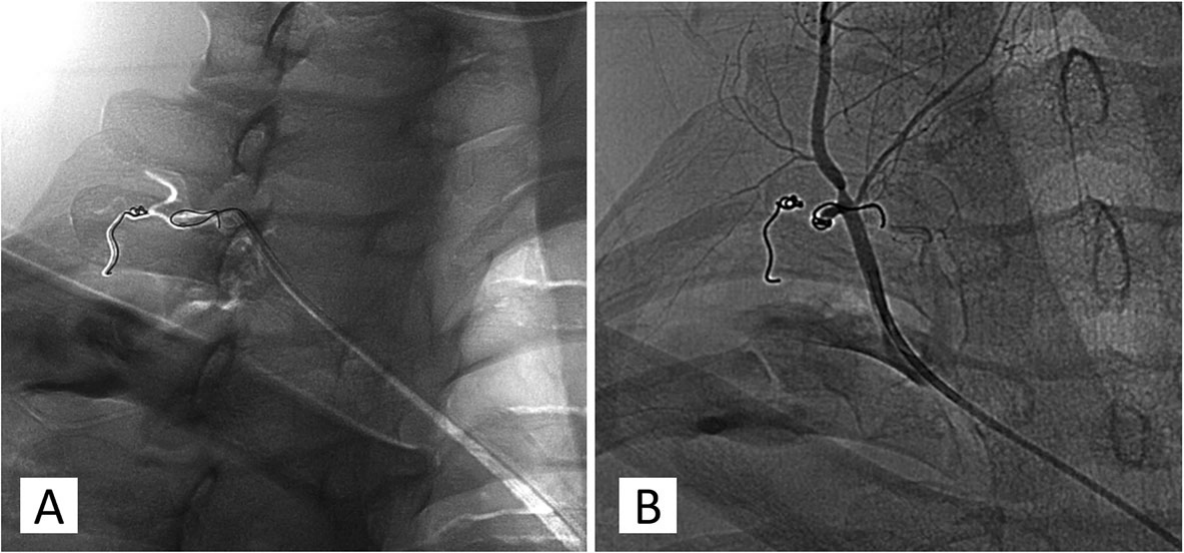
Embolisation with spirals. The first Fig. (**a**) shows an angiogram of the tortuous course of the bleeding vessels. The micro-catheter was inserted over the branch supplying the spinal cord in order to obtain a selective embolisation of the blood leaking branches with two micro-coils. The second angiographic image (**b**) demonstrated the closure of the haemorrhagic vessels and the patency of the radiculo-medullary spinal branch. The typical longitudinal course of the anterior spinal artery is shown in the right frame, where it runs in parallel to the spinal processes of the vertebrae

Finally, the patient was transferred to the angiographic room for the treatment of the disrupted vessel. The procedure consisted in a right transfemoral artery access. Digital Subtractive Angiography was performed to assess the thoracic aorta and the right posterior intercostal arteries, resulting negative for active bleeding. The subclavian artery and its branches were evaluated, showing a consistent and faint contrast media leaking in the vascular field of the thyrocervical trunk (Fig. 3). After catheterisation, this latter vessel showed to be the source of the suspected bleeding. A radicular-medullary spinal artery arising from the thyrocervical trunk was also pointed out by means of the injection of contrast media. The presence of the mentioned spinal cord feeding branch suggested not to use the microparticles in order to avoid its accidental embolisation, requiring instead to slide further the catheter tip. Through a micro-catheter, two micro-coils (3x20mm) were inserted to stop the haemorrhagic flow. At the angiographic control any bleeding or collateral supply to the haematoma emerged (Fig. 4). The embolised branches presented a tortuous course that led to a complicated endovascular manoeuvre, which forced the last coil to make a turn-around from the inserting direction.

The patient was hospitalised and the lower haemoglobin levels were stationary (Serum Haemoglobin level = 7,6 mg/dL). A CT scanning was performed after 3 days from the endovascular treatment and it did not show any sign of contrast media blushing.

## Conclusion

Currently, juggling is spreading as a common practice, often considered as a new training activity, involving a high risk of trauma and unexpected complications. As the juggler does not show how hard his work is, thanks to a conscious control of the danger, a good interventional radiologist needs to foresee and avoid the risks, by having confidence in managing them.

The endovascular treatment of the thyrocervical trunk branches may have difficulties due to the course of the vessels and the eventual presence of spinal cord supplying artery contraindicates the use of microparticles. CT angiography in our patient missed describing the presence of the spinal collateral branch and cannot be considered reliable in revealing anomalous connections with other vessels: a further study of the vascular anatomy during angiography is mandatory.

In our experience a selective approach is mandatory in order to preserve the integrity of the axial nervous system. Therefore, during every interventional procedure, the operator should be prepared to choose the best technique to control the bleeding and prevent the predictable complications aiming for the best outcome for the patient.

To the best of our knowledge, this paper is the first describing the procedure of embolisation for a bleeding of the thyrocervical trunk after a blunt trauma inserting the distal extremity of the coil along the bleeding vessel, like a wire, to permit to the proximal closure of the vessel itself and preventing from the risk of spinal infarct in case of microparticles use.

## Data Availability

Data sharing is not applicable to this article, because no datasets were generated or analyzed during the current study.

## Abbreviation

CT: Computed Tomography
